# Long-Term Opioid Therapy in Spine Center Outpatients: Protocol for the Spinal Pain Opioid Cohort (SPOC) Study

**DOI:** 10.2196/21380

**Published:** 2020-08-19

**Authors:** Claus Manniche, Lonny Stokholm, Sophie L Ravn, Tonny E Andersen, Lars PA Brandt, Katrine H Rubin, Berit Schiøttz-Christensen, Lars L Andersen, Søren G Skousgaard

**Affiliations:** 1 Department of Occupational and Environmental Medicine Institute of Clinical Research University of Southern Denmark Odense Denmark; 2 Open Patient Data Explorative Network Department of Clinical Research University of Southern Denmark and Odense University Hospital Odense Denmark; 3 Department of Psychology University of Southern Denmark Odense Denmark; 4 Specialized Hospital for Polio and Accident Victims Roedovre Denmark; 5 Department of Occupational and Environmental Medicine Odense University Hospital Odense Denmark; 6 Institute of Clinical Research University of Southern Denmark Odense Denmark; 7 Spine Centre of Southern Denmark Middlefart Hospital-Lillebælt Hospital Middelfart Denmark; 8 University of Southern Denmark Odense Denmark; 9 National Research Centre for the Working Environment Copenhagen Denmark

**Keywords:** spine, lower back pain, cohort study, opioid, long-term opioid therapy (LTOT), therapy, pain, protocol, patient data, outpatient

## Abstract

**Background:**

Spinal pain is the leading cause of patient-years lived with chronic pain and disability worldwide. Although opioids are well documented as an effective short-term pain-relieving medication, more than a few weeks of treatment may result in a diminishing clinical effect as well as the development of addictive behavior. Despite recognition of opioid addiction in pain patients as a major problem commonly experienced in the clinic, no reference material exists on the scope of long-term problems in novel opioid users and the link to clinical outcomes.

**Objective:**

The main aims of this study are to describe baseline and follow-up characteristics of the Spinal Pain Opioid Cohort (SPOC), to evaluate the general use of opioids in spinal pain when an acute pain episode occurs, and to demonstrate the prevalence of long-term opioid therapy (LTOT).

**Methods:**

Prospective clinical registry data were collected from an outpatient spine center setting during 2012-2013 including patients with a new spinal pain episode lasting for more than 2 months, aged between 18 and 65 years who had their first outpatient visit in the center. Variables include demographics, clinical data collected in SpineData, the Danish National Patient Register, and The Danish National Prescription Registry. The primary outcome parameter is long-term prescription opioid use registered from 4 years before the first spine center visit to 5 years after.

**Results:**

This is an ongoing survey. It is estimated that more than 8000 patients fulfill the SPOC inclusion criteria. In 2019, we began the intellectual process of identifying the most relevant supplementary data available from the wide range of existing national registries available in Denmark. We have now begun merging SpineData with relevant opioid data from Danish national registers and will continue to extract data up to 2021-2022. We will also be looking at data regarding somatic or psychiatric hospitalization patterns, patient usage of health care resources, as well as their working status and disability pensions.

**Conclusions:**

To our knowledge, this survey will be the first to document the scope of long-term problems regarding LTOT and opioid addiction following new spinal pain episodes and comparing descriptive follow-up data between substance users and nonusers.

**Trial Registration:**

ISRCTN Registry ISRCTN69685117; http://www.isrctn.com/ISRCTN69685117

**International Registered Report Identifier (IRRID):**

DERR1-10.2196/21380

## Introduction

Spinal pain is the leading worldwide cause of patient-years lived with chronic pain and disability [1,2]. The impact on resources related to diagnostic procedures and treatment, including rehabilitative activities, is enormous [3,4]. Despite all of the resources allocated to the management of patients with spinal pain, the incidence of disability problems is projected to increase over the coming decades [5]. One of the most widely prescribed treatment forms is medical analgesics [6]. Opioids are well documented as an effective short-term pain-relieving medication [7,8] and are frequently prescribed in North America and most European countries [8-12]. However, more than a few weeks of treatment may well result in a diminishing clinical effect, risk of opioid-generated hyperalgesia that worsens the pain, as well as the development of addictive behavior [8]. Additionally, long-term opioid therapy (LTOT) may also result in a long series of somatic and psychological side effects such as depression, anxiety, and pain catastrophizing, as well as generally reduced physical activity [13-16]. Moreover, long-term usage of opioids is associated with lower rates of return to work in injured workers and the risk of social isolation issues [8]. Adding to the general health risk of prescribing opioids is the possible harm of adverse selection [15] and the fact that personal premorbid psychological disorders may impact the individual prognostic course following the first spinal pain episode [17].

Despite the increasing long-term dependence risk of change in addictive behavior associated with opioid treatment [18], most published spinal pain cohorts do not include follow-up data beyond 1 year [19]. Additionally, only a few studies have focused on spinal pain patients [8]. Thus, long-term studies are necessary to develop benchmark reference material documenting the scope of potential problems of opioid addiction to target preventive strategies more effectively.

The aim of this prospective Spinal Pain Opioid Cohort (SPOC) research program is to collate relevant individual patient data over a decade to illuminate both the overall group data developments as well as developments relating to individual usage of different opioids during this period, and to correlate these findings with the individual patient’s physical, psychological, and social data over 10 years. In Denmark, a large number of different national registers are available, and the present data will provide the opportunity to assess different long-term effects of opioid use.

## Methods

### Study Design

This is a longitudinal cohort study based on prospectively collected data as part of routine daily clinical practice in The Spine Center of Southern Denmark during 2012-2013. This outpatient secondary care department is a unit in a public hospital with a geographic catchment area of approximately 1.2 million inhabitants [20]. The department applies multidisciplinary assessments of patients with spinal pain after a referral from general practitioners, chiropractors, and medical specialists in primary care.

**Figure 1:**
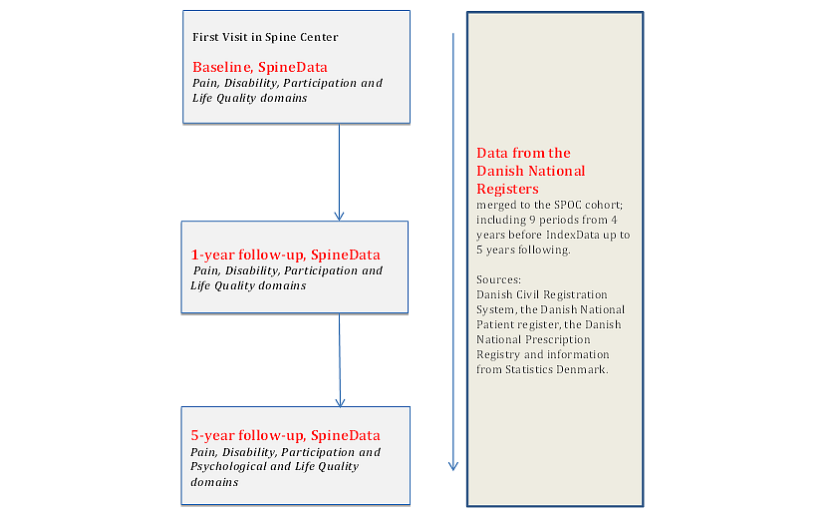
Spinal Pain Opioid Cohort (SPOC) flowchart.

Initial data are collected in the Spine Center’s electronic clinical registry, named the SpineData database, at the date of the first visit (Regional Ethics Committee Project ID: S-200112000-29) [20]. Patients answered a comprehensive self-reported baseline questionnaire on a touch screen in the waiting area before their first consultation. The patient could identify the area of pain on the screen and choose between neck pain, midback pain, and lower back pain. The patient’s initial choice of which area of the spine was giving them the most trouble determined which anatomical subgroup they were enrolled in. All follow-up data have been gathered using an internet-based version of the SpineData database and patients are enrolled in the analyses by linking data from the SPOC with data from the Danish national registers. An overview of the data collection and subgroups for the SPOC is provided in Figure 1.

### Study Population

Patients aged between 18 and 65 years who had a new pain episode for more than 2 months and had their first outpatient visit at the Spine Center between November 1, 2012 and December 31, 2013, with no indications for acute spinal surgery intervention, are enrolled in the SPOC. Patient symptoms indicating serious systemic diseases such as cancer, infections, or inflammatory spondylopathies are excluded a priori. An individual patient is included in the SPOC if the baseline questionnaires has no missing data related to pain intensity. 

### Variables and Domains

In accordance with the biopsychosocial model of health, information is collected across a set of broad health domains of pain, activity limitation, work participation, psychological factors, physical impairment, and contextual factors. Wherever possible, the choice of questions and questionnaires is based on evidence of their role in the diagnosis, prognosis, or treatment of spinal pain. The questions vary across the three spinal regions of principal complaint: neck pain, midback pain, and lower back pain [20].

#### Pain Domain

Patient-reported questions include a main pain chart (current pain) and other pain charts (any additional areas of pain during the previous 2 weeks), the onset date of pain, any previous lower back pain or radiating episodes, pain intensity (current, typical, and worst in last 14 days), extremity pain intensity (current, typical, and worst in last 14 days), number of days per week with pain, cause of or reason for onset, morning stiffness, diurnal variation, movement-related pain, activity-related pain, the effect of physical rest on pain, and pain easily aggravated by movement [21].

#### Activity Limitation Domain

Patient-reported questions include the 23-item Roland-Morris Disability Questionnaire for lower back pain [22,23] and the Neck Disability Index for midback or neck pain [24].

#### Participation Domain

Patient-reported questions include type of employment, whether on sick leave due to back pain at any time in the last 3 months and for how long, expectation of working in 6 months, physically strenuous work, monotonous work, and work satisfaction [20].

#### Psychological Domain

Levels of depressive symptoms and anxiety are measured with the Hospital Anxiety and Depression Scale (HADS) [25]. The HADS was originally constructed to detect anxiety and depression in nonpsychiatric medical patients. It was subsequently shown to be useful as a “case finder” in other populations, and it is a well-validated questionnaire with good psychometric properties [25]. The depression subscale consists of 7 items related to depression and the anxiety subscale consists of 7 items related to anxiety [25]. Symptom levels on both subscales are measured on a 4-point Likert scale for each item, resulting in symptoms ranging from 0 to 21 for each subscale, with a high score indicating high levels of depression and anxiety.

Exposure to traumatic events is assessed by a modified version of the Life Events Checklist-5 (LEC-5) [26]. Among a list of a large number of possible traumatic events, patients are asked whether they have witnessed or been directly exposed to any of the events. Examples of traumatic events listed are natural disasters, accidents, assaults, or life-threatening illnesses. Results are reported as exposure to the number of trauma types.

The International Trauma Questionnaire (ITQ) [27] can be used to assess symptoms of posttraumatic stress disorder (PTSD) related to the index trauma as identified on the LEC-5. The ITQ is a 6-item questionnaire assessing PTSD symptoms according to the International Classification of Diseases (ICD)-11 criteria on three clusters of reexperiencing, avoidance, and hyperarousal. Each item is rated on a 5-point Likert scale ranging from 0 (not at all) to 4 (extremely) indicating how much each symptom has bothered the respondent in the past month. A probable PTSD diagnosis can be calculated according to the ICD-11 criteria if the respondent endorses at least one symptom on each PTSD cluster, as indicated by a score2.

Attachment security is measured on the Experiences in Close Relationship Scale-Short Form (ECR-S) [28]. The ECR-S is a 12-item self-report instrument that measures attachment-related anxiety and avoidance. Participants are asked to think about how they generally experience close relationships and to rate the extent to which each item accurately describes their feelings in such relationships using a 7-point Likert scale ranging from 1 (not at all) to 7 (very much). Six items measure attachment-related anxiety (eg, “I need a lot of reassurance that I am loved by my partner”) and 6 items measure avoidance (eg, “I try to avoid getting too close to my partner”).

#### Contextual Factors Domain

Patient-reported questions include height, weight, previous back surgery, prolonged corticosteroid use, exposure to prolonged mechanical vibration, handedness, level of recreational physical activity, allergies, cigarette use, alcohol consumption, serious lung disease, heart disease, or cancer, which are generated from SpineData [20].

#### Quality of Life

Quality of life is measured using a validated and recognized Danish self-perceived general health scale inspired by the EuroQoL Health Measure Thermometer (0-100) [20,29].

### Follow-Up Questionnaires

All patients are invited to complete two electronically provided follow-up questionnaires that contain representative questions from the baseline patient questionnaires. The first email follow-up questionnaire takes place 12 months after the date of the initial consultation. The second email-based follow-up questionnaires are sent to the patient as a 5-year follow up. All of the patients received an electronic questionnaire and have been asked to complete the questionnaire and return it via email. If a patient did not respond, they were contacted 2 more times after the first email communication.

### Data From National Registers

Linking data from the SPOC with data from the Danish national registers is possible using the Danish Identity Number (known as the CPR number) assigned to all citizens in Denmark at birth or immigration. We link the SPOC with information from the Danish Civil Registration System (CRS) [30], Danish National Patient Register (DNPR) [31], Danish National Prescription Registry (NPR), [32] and information from Statistics Denmark.

The DNPR contains information on all inpatient and outpatient visits as well as emergency room visits in Denmark. From the DNPR we extract information on the patient’s comorbidities including 5 years ending on the date of the first visit in the Spine Center and using the diagnostic codes according to the disease-specific ICD-10 [33]. Data in the NPR [32] are collected on an individual level and include all drugs dispensed by prescription at community pharmacies. For this study, information on prescribed drugs is available from 2007 to June 30, 2018.

The study also provides an opportunity to link the data to the many different national registers available in Denmark [34], and therefore provides the opportunity for further national and international collaboration on the long-term effects of opioid use (Figure 1).

### Comorbidity

Based on the diagnostic codes (ICD-10) from DNPR, we calculate comorbidity among the individuals at their first visit to the Spine Center. We use the Charlson Comorbidity Index to classify comorbidity among the individuals, which is based on 19 comorbid conditions [33,35].

### Opioid-Related Variables

#### Classification of Opioids

The prescribed and dispensed medications from the NPR are classified according to the Anatomical Therapeutic Chemical (ATC) classification system [36]. We extract dispensed opioids from the NPR using the following ATC codes: morphine (N02AA01), hydromorphone (N02AA03), oxycodone (N02AA05), oxycodone combination (N02AA55), ketobemidone (N02AB01), pethidine (N02AB02), fentanyl (N02AB03), dextropropoxyphene (N02AC04), buprenorphine (N02AE01), ketobemidone (N02AG02), tramadol (N02AX02), tapentadol (N02AX06), codeine with paracetamol (N02AJ06), codeine with acetylsalicylic acid (N02AJ07), codeine with ibuprofen (N02AJ08), and codeine (N02AJ09).

#### Time Intervals for Prescribed Opioids

The Spine Center referral guidelines generally state that patients should not be referred to the center before having experienced pain for at least 2 months (mean 4.5 months) and for no longer than 12 months since symptom onset. As a consequence, for the present survey, we presume that the most intense pain period in the referred patients typically will begin and be concluded in the 364-day period minus 182 days (IndexDate) and up to 182 days following the day of the first visit in the Spine Center. In other words, the definition of the IndexDate is the date of the first visit to the Spine Center minus 182 days.

From the IndexDate, we define a total of 9 observation intervals for a single patient, including 4 intervals before and 5 intervals after the IndexDate. Each interval consists of a period of 364 days. The numbers of dispensed prescriptions are counted in each interval.

#### Definition of Subgroups

**Figure 2:**
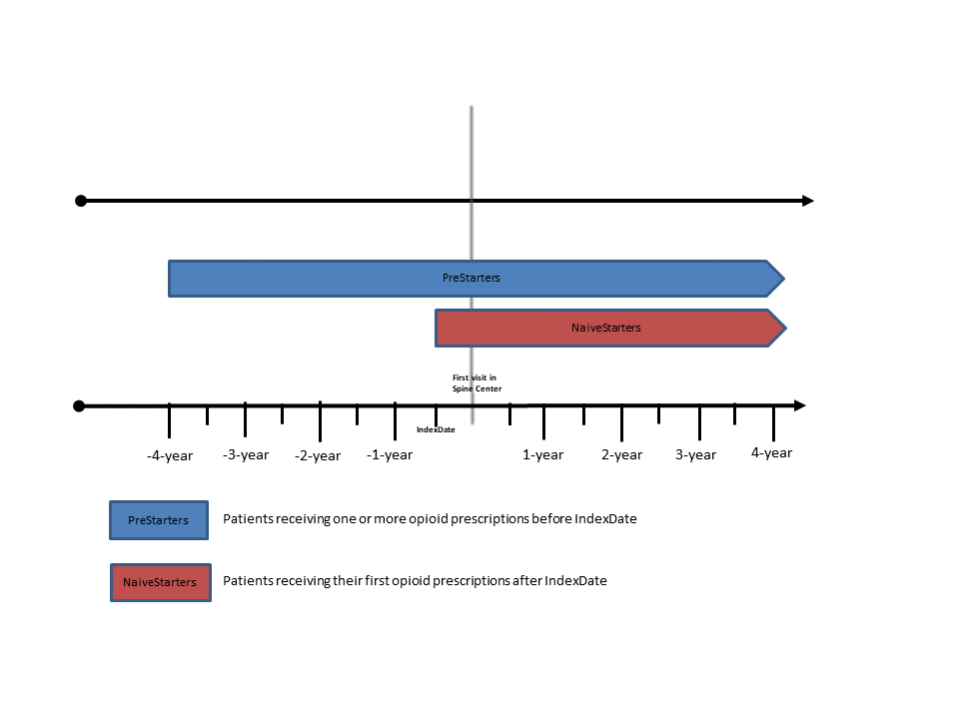
Spinal Pain Opioid Cohort subgroups receiving opioid prescriptions.

For most of the analyses, patients in the SPOC will be separated into the following 3 subgroups: (1) patients who do not receive any opioid prescriptions at any observation point (nonopioid users); (2) patients who have their very first opioid prescription following IndexDate (NaiveStarters); and patients who had one or more opioid prescriptions in the intervals before IndexDate (PreStarters). The separation between PreStarters and NaiveStarters allows us to differentiate patients with former spine pain episodes and related use of opioids before the IndexDate (Figure 2).

#### Primary Outcome Parameter: LTOT

This study’s primary outcome parameter was inspired by “The Copenhagen Criteria” definitions and methods to register LTOT [11]. In our study, 6 or more opioid prescriptions in a single 1-year interval fulfill the LTOT criteria in that interval. The prevalence for patients fulfilling LTOT in the respective subgroups is calculated for all 9 364-day intervals. Additionally, the prevalence of receiving at least one prescription in a single interval will be evaluated.

### Statistical Modeling

Baseline, and 1- and 5-year follow-up characteristics of the SPOC will be reported either as proportions or median values with IQR. We use the median and IQR for continuous variables that are not normally distributed. Differences between the level of comorbidity according to the Charlson comorbidity index [33,35] are also calculated. Possible statistically significant differences between the subgroups will be calculated using the Chi-square test. Differences are considered to be statistically significant at *P*<.05. Analyses are performed using STATA version 14.2 (StataCorp, College Station, TX, USA).

### Ethics

Ethics approval for the collection and use of these data for quality assurance and research purposes has been provided by the Scientific Ethics Committee of the Region of Southern Denmark (project ID S-200112000-29). The database is also registered with the Danish Data Protection Agency (2008-58-0035). All patients have been invited to give two types of written informed consent. The first is for their patient data to be used for quality assurance and research purposes, including publications of anonymized group-level data, and the second is for the Spine Center to contact them requesting the completion of follow-up questionnaires.

## Results

The project is ongoing. The gathering of questionnaire data among SPOC patients began in 2012. From 2013 to 2019, 1-year and 5-year follow-up data were collected and stored in the Spine Center’s SpineData database. It is estimated that more than 8000 patients fulfill the SPOC inclusion criteria. In 2019, we began the intellectual process of identifying the most relevant supplementary data available from the wide range of existing national registries available in Denmark. We have now begun merging SpineData with relevant opioid data from the NPR and will continue to extract data from this register as well as other relevant Danish national registers up to 2021-2022. We will be looking at data regarding somatic or psychiatric hospitalization patterns, patient usage of health care resources, as well as their working status and disability pensions. We have plans to run a 10-year follow-up study.

## Discussion

This survey will be the first to document the scope of long-term problems regarding LTOT and opioid addiction following new spinal pain episodes and comparing opioid usage in PreStarters and NaiveStarters. Usage will be registered from 4 years before the new pain episode and for 5 years afterward. In parallel, we will be able to present continuously descriptive follow-up data from the SPOC.

Previously published data indicate that Danes have relatively high utilization of opioids in patient groups experiencing benign pain in the musculoskeletal system [8,11]. A reasonable prediction of the study results is that a relatively high percentage of SPOC patients will continue to use opioids for several years, including the last follow-up period and probably for the rest of their lives for some patients. It will be interesting to carry out a subgroup analysis between NaiveStarters and PreStarters. Reasonably more individuals in the latter group are at risk to have LTOT status compared to NaiveStarters. In addition, it will be relevant to carry out future analyses of the impact of social and psychological elements on the SPOC general prognosis and LTOT prevalence [8,15,17].

The structure of this study involves both methodological strengths and limitations. One of the absolute strengths in this study is the possible high number of included patients, all of whom experience pain from the same anatomical structures. Additionally, by using the links to national databases during the entire study period, we will be able to obtain a complete dataset for all of the patients regarding their ongoing use of opioids. All of the patients are included from the same regional health system and only one health care entity; in other words, a relatively homogeneous group of patients will be included in the study. A limitation is that we will not be able to gather general data regarding the stoppage of opioid utilization due to side effects or the frequency of side effects.

From the long-term collection of relevant individual patient data, it will be possible to illuminate both the overall group data developments as well as long-term developments relating to individual usage of different opioids and to correlate these findings with the individual patient’s physical, psychological, and social data. Results obtained in this study may help to identify a subset of patients with chronic pain for whom long-term opioid use is both safe and effective [8,37,38].

We expect that SPOC data in the future will contribute research information qualifying the debate about the importance of the clinician undertaking a very thorough patient selection process before prescribing the very first opioid prescription and to provide patients with a solid understanding of the risks of developing addictive behavior and other potential side effects related to LTOT.

## Abbreviations

ATC: Anatomical Therapeutic Chemical classification system
CRS: Danish Civil Registration System
DNPR: Danish National Patient register
ECR-S: Experiences in Close Relationship Scale-Short Form
HADS: Hospital Anxiety and Depression Scale
ICD: International Classification of Diseases
ITQ: International Trauma Questionnaire
LEC-5: Life Events Checklist-5
LTOT: Long-Term Opioid Therapy
NPR: Danish National Prescription Registry
PTSD: posttraumatic stress disorder
SPOC: Spinal Pain Opioid Cohort
